# *Otaciliakhezu* sp. nov., a new troglobitic spider (Araneae, Phrurolithidae) from Guangxi, China

**DOI:** 10.3897/BDJ.12.e126716

**Published:** 2024-06-13

**Authors:** Yejie Lin, Haifeng Chen, Xihao Wang, Shuqiang Li

**Affiliations:** 1 Hebei Key Laboratory of Animal Diversity, College of Life Science, Langfang Normal University, Langfang, Hebei 065000, China Hebei Key Laboratory of Animal Diversity, College of Life Science, Langfang Normal University Langfang, Hebei 065000 China; 2 Jinan Licheng No.2 High School, Jinan 250109, China Jinan Licheng No.2 High School Jinan 250109 China; 3 Institute of Zoology, Chinese Academy of sciences, Beijing 100101, China Institute of Zoology, Chinese Academy of sciences Beijing 100101 China

**Keywords:** Asia, diagnosis, morphology, spider, taxonomy

## Abstract

**Background:**

Only two *Otacilia* Thorell, 1897 species with troglobitic characteristics have been recorded from Laos and no records of troglobitic *Otacilia* species from China.

**New information:**

A new troglobitic species is reported from Guangxi, China: *Otaciliakhezu* Lin & Li, **sp. nov.** (♂♀). Photos and morphological descriptions of the new species are presented; the type specimens of the new species are deposited in the Institute of Zoology, Chinese Academy of Sciences (IZCAS), Beijing.

## Introduction

*Otacilia* Thorell, 1897 is a genus of spiders belonging to the family Phrurolithidae Banks, 1892, commonly known as the guardstone spiders. *Otacilia* are distributed in East and Southeast Asia, with notable populations found in China. Their geographical range reflects their adaptability to diverse habitats, ranging from forests and grasslands to urban environments (Liu et al. 2022, Mu and Zhang 2023). This broad distribution underscores the importance of studying *Otacilia* spiders to gain insights into ecosystem dynamics and biodiversity conservation efforts.

At present, 137 species are known worldwide, of which 115 species are distributed in China, with only nine females and six males being known. Research in China has also been rapid, with only three species known before 2000. In past two years, totally 47 new species have been described. (Liu et al. 2022, Lu et al. 2022, Zhao et al. 2022, Zhang et al. 2023, Luo and Li 2024, Li and Lin 2024, World Spider Catalog 2024).

**Figure 1.**
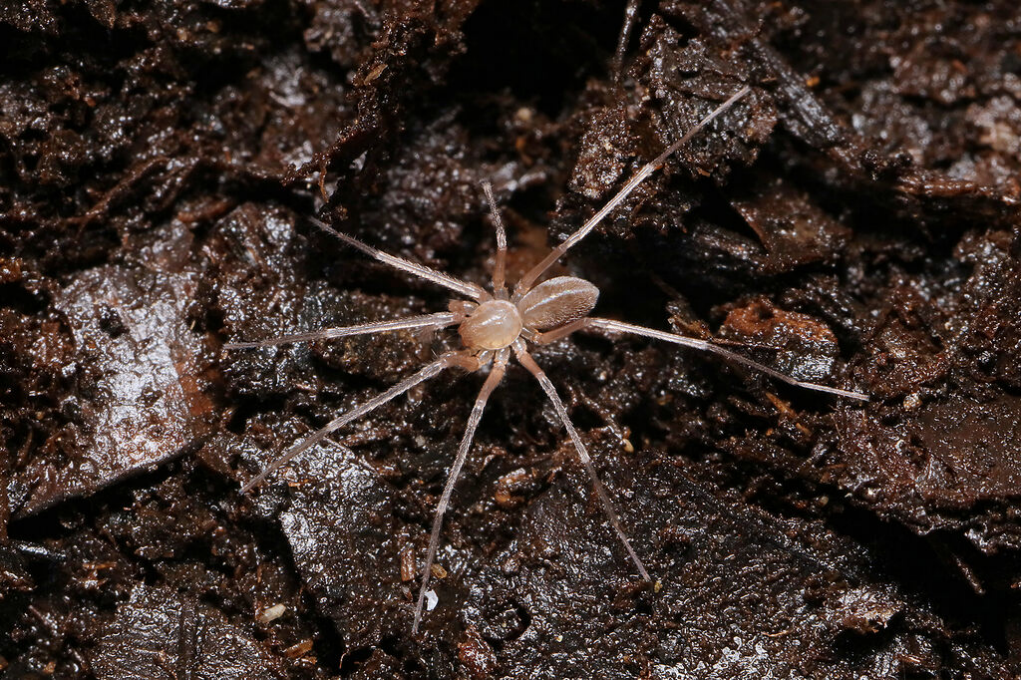
*Otaciliakhezu* sp. nov., juvenile, in life. Photo: Shanmi Zheng.

Only two species with troglobitic characteristics have been recorded from Laos: *O.saszykaska* Jäger, 2022 and *O.tham* Jäger, 2022, all of them with strong reduction of eye and body pigments (Jäger 2022). Hitherto, there have been no records of troglobitic *Otacilia* species from China. In this paper, we report a new troglobitic *Otacilia* species with loss of eyes from Guangxi, China (Fig. 1).

## Materials and methods

All specimens were preserved in 80% ethanol. The spermathecae were cleared in trypsin enzyme solution to dissolve non-chitinous tissues. Specimens were examined under a LEICA M205C stereomicroscope. Photomicrographs were taken with an Olympus C7070 zoom digital camera (7.1 megapixels). Laboratory habitus photographs were taken with a Sony A7RIV digital camera equipped with a Sony FE 90 mm Goss lens. Photos were stacked with Helicon Focus (Version 7.6.1) or Zerene Stacker (Version 1.04) and processed in Adobe Photoshop CC2022.

All measurements are in millimetres and were obtained with an Olympus SZX16 stereomicroscope with a Zongyuan CCD industrial camera. All measurements of body lengths do not include the chelicerae. Eye sizes are measured as the maximum diameter from either the dorsal or frontal view. Leg measurements are given as follows: total length (femur, patella+tibia, metatarsus, tarsus), the terminology used in the text and figures following Mu and Zhang (2023).

Abbreviations: B—bursa; CD—copulatory duct; CO—copulatory opening; CT—connecting tube; DTA—dorsal tibial apophysis; E—embolus; FA—femoral apophysis; FD—fertilisation duct; GA—glandular appendage; RTA—retrolateral tibial apophysis; SD—sperm duct; S—spermathecae; TA—tegular apophysis.

Types from the current study are deposited in the Institute of Zoology, Chinese Academy of Sciences in Beijing (**IZCAS**).

## Taxon treatments

### Otacilia khezu

Lin & Li sp. nov.

5A796256-4291-5465-9D1A-1B8CAC681986

18362B5A-4389-4C4B-8EB1-DFA14605E861

#### Materials

- **Type status:** Holotype. **Occurrence:** recordedBy: Haolin Mo and Shanmi Zheng; sex: male; occurrenceID: E52E789E-3C2D-57AD-BA5E-258D96D79029; **Taxon:** scientificName: *Otaciliakhezu* sp. nov.; **Location:** country: China; stateProvince: Guangxi; county: Du'an Yao Autonomous County; locality: Jingsheng Town, Unnamed Cave; verbatimElevation: 610 m; decimalLatitude: 23.9732; decimalLongitude: 108.2920; **Identification:** identificationID: IZCAS-Ar43962; identifiedBy: Yejie Lin; dateIdentified: 2024; **Event:** year: 2024; month: 2; day: 22; habitat: Cave
- **Type status:** Paratype. **Occurrence:** recordedBy: Haolin Mo and Shanmi Zheng; sex: female; occurrenceID: 7C5E8DDA-FD40-560D-81D6-33CD4E187ED5; **Taxon:** scientificName: *Otaciliakhezu* sp. nov.; **Location:** country: China; stateProvince: Guangxi; county: Du'an Yao Autonomous County; locality: Jingsheng Town, Unnamed Cave; verbatimElevation: 610 m; decimalLatitude: 23.9732; decimalLongitude: 108.2920; **Identification:** identificationID: IZCAS-Ar43963; identifiedBy: Yejie Lin; dateIdentified: 2024; **Event:** year: 2024; month: 2; day: 22; habitat: Cave

#### Description

**Male (holotype)**. Total length 2.77; carapace 1.37 long, 1.10 wide, opisthosoma 1.43 long, 0.91 wide. Eyes absent. Chelicerae with two promarginal and two retromarginal teeth. Leg measurements: I 8.58 (2.17, 3.07, 2.14, 1.20), II 7.59 (1.91, 2.60, 1.82, 1.26), III 7.39 (1.84, 2.20, 1.94, 1.41), IV 9.54 (2.43, 2.84, 2.57, 1.70). Leg spination: I femur 5pv, 2d, tibia 9-9v, metatarsus 5-5v; II femur 3pv, 2d, tibia 9-9v, metatarsus 5-5v, III femur 1d, IV femur 1d.

**Colouration** (Fig. 4A). Carapace pale yellow, without any pattern, cover with separated long hair. Chelicerae yellow. Endites and labium yellow. Sternum paler yellow. Legs paler yellow without any pattern. Opisthosoma oval, grey, dorsal scutum absent. Spinnerets grey.

**Figure 2.**
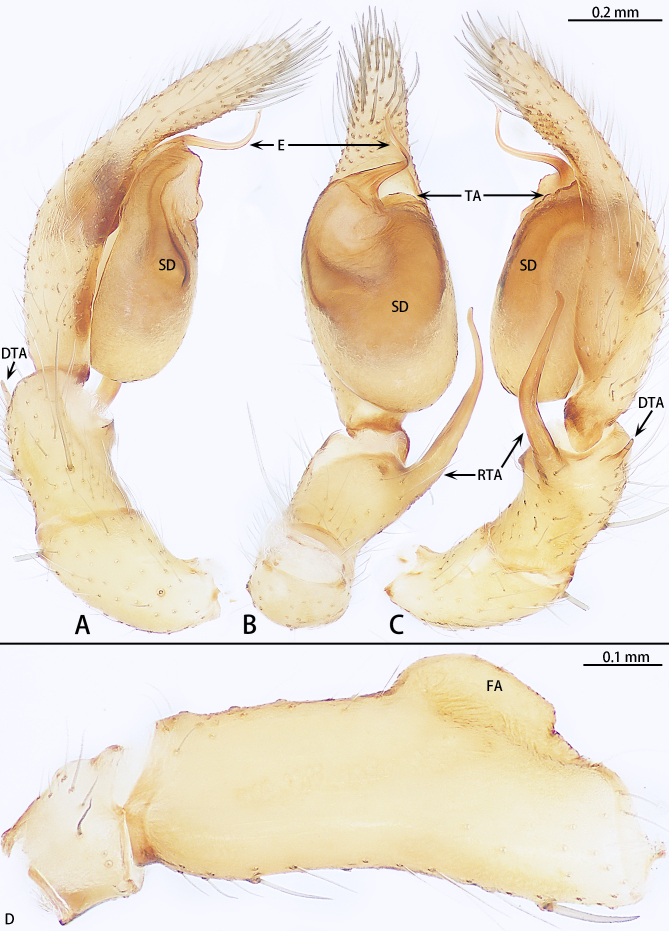
*Otaciliakhezu* sp. nov, holotype male. **A** Palp, prolateral view; **B** Same, ventral view; **C** Same, retrolateral view; **D** Palp femur, Retrolateral view. Abbreviations: DTA dorsal tibial apophysis; E embolus; FA femoral apophysis; RTA retrolateral tibial apophysis; SD sperm duct; TA tegular apophysis.

**Palp** (Fig. 2A–D). Femur distally with an inflated hump on ventral side and a retrolateral concavity, almost as long as the width of patella, with groove. Patella longer than wide. Retrolateral tibial apophysis slightly curved, slender, almost as long as bulb. Dorsal tibial apophysis short, only one fifth of retrolateral tibial apophysis, terminus needle-shaped. Cymbium 2.5 times longer than wide. Bulb oval. Sperm duct U-shaped. Tegular apophysis semicircular. Embolus spirally, curved.

**Female (paratype Ar43963)**. Total length 2.79; carapace 1.42 long, 1.19 wide, opisthosoma 1.43 long, 0.78 wide. Eyes absent. Chelicerae with two promarginal and two retromarginal teeth. Leg measurements: I 8.02 (2.12, 2.98, 1.86, 1.06), II 7.18 (1.87, 2.56, 1.64, 1.11), III 7.21 (1.85, 2.21, 1.81, 1.34), IV lost. Leg spination: I femur 5pv, 2d, tibia 10-10v, metatarsus 5-5v; II femur 3pv, 2d, tibia 9-9v, metatarsus 5-5v, III femur 1d, IV absent.

**Colouration** (Fig. 4B). Similar to that of male.

**Figure 3.**
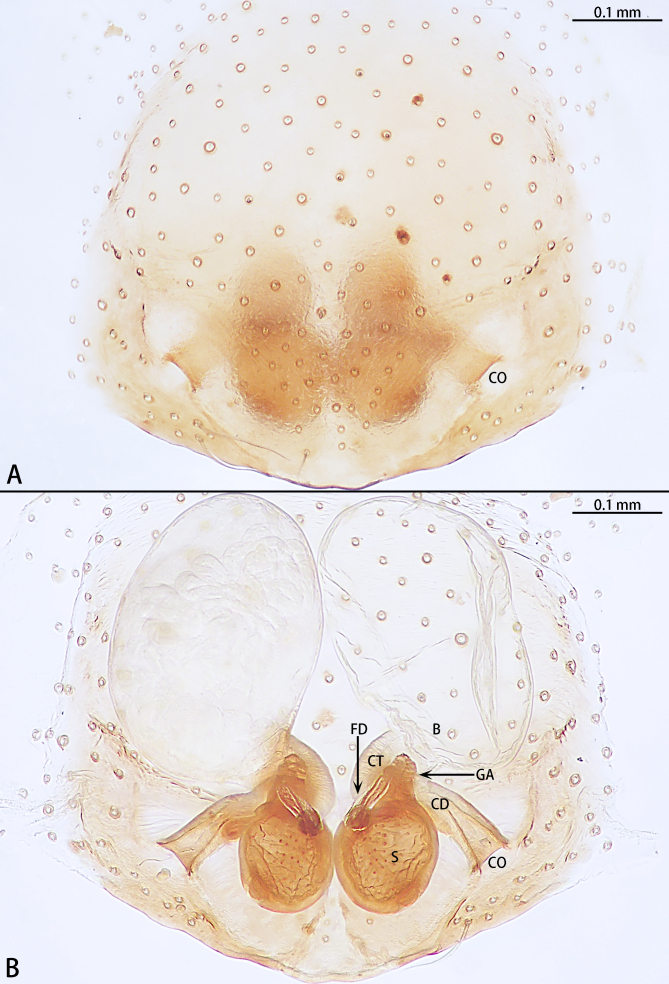
*Otaciliakhezu* sp. nov., paratype female. **A** Epigyne, ventral view; **B** Vulva, dorsal view. Abbreviations: B bursa; CD copulatory duct; CO copulatory opening; CT connecting tube; FD fertilisation duct; GA glandular appendage; S spermathecae.

**Epigyne** (Fig. 3A and B). Epigynal plate oval. Copulatory openings oval, separated, at the middle of the epigynal plate. Copulatory ducts slightly curved, almost as long as spermathecae, almost two times longer than connecting tubes. Connecting tubes slightly curved. Bursae large, almost oval, 1.5 times wider than long, covering nearly half of epigynal plate. Glandular appendages obvious, located at the spermathecae anteriorly. Spermathecae oval. Fertilisation duct (left) directed at 1 o’clock position from spermathecae.

#### Diagnosis

**Figure 4.**
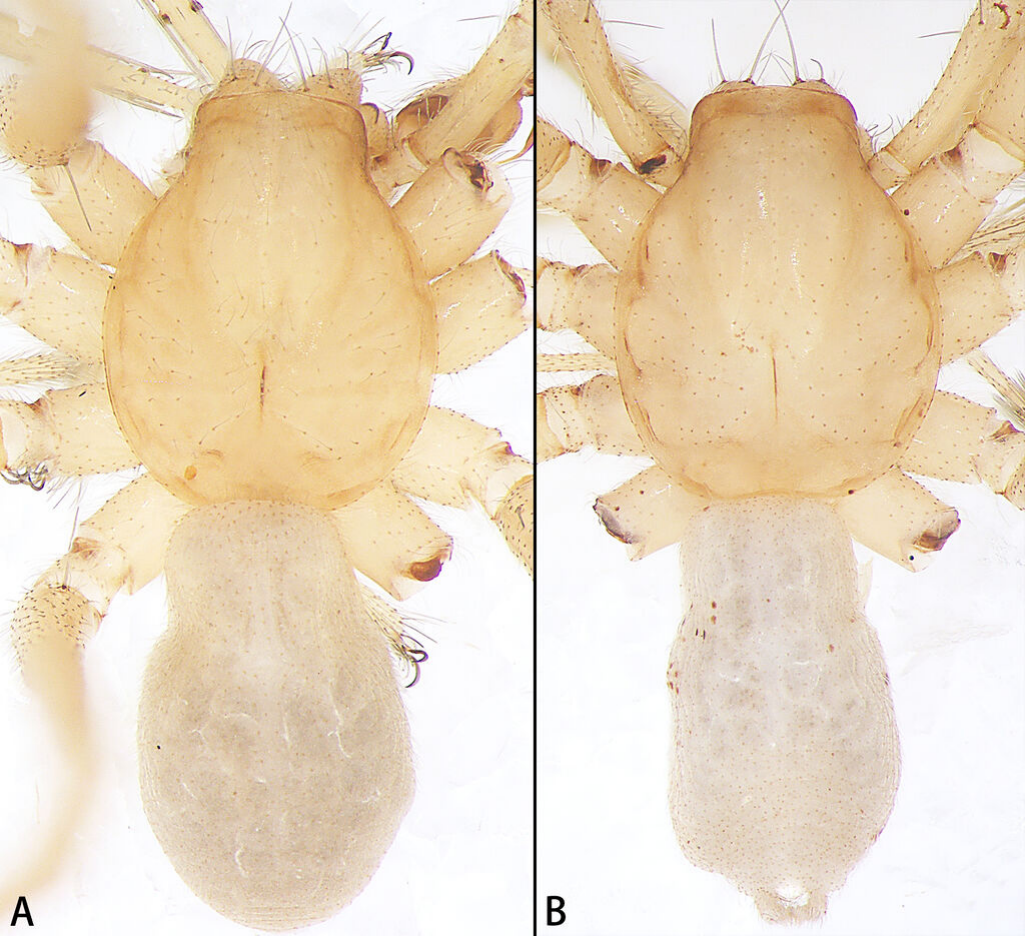
Habitus of *Otaciliakhezu* sp. nov., dorsal view. **A** holotype male; **B** paratype female.

The new species can be distinguished from other colleagues by the absence of eyes and dorsal scutum (Fig. 4).

In male palp, this species with the spiral embolus, slender retrolateral tibial apophysis almost as long as bulb and the needle-shaped dorsal tibial apophysis (Fig. 2A–C). The female is similar to *O.allomanubrium* Mu & Zhang, 2023 by the separated copulatory openings and glandular appendage directed anteriorly (see Mu and Zhang (2023), figs. 20E, F; figs. 21D and E). However, the female of this new species can be distinguished from *O.allomanubrium* by the copulatory ducts almost as long as spermathecae (Fig. 3B) (copulatory ducts not obvious) and glandular appendage located at spermathecae (Fig. 3B) (at connecting tube).

#### Etymology

The species is named after *khezu*; a kind of blind flying wyvern first appearing in Monster Hunter, noun in apposition.

#### Distribution

**Figure 5.**
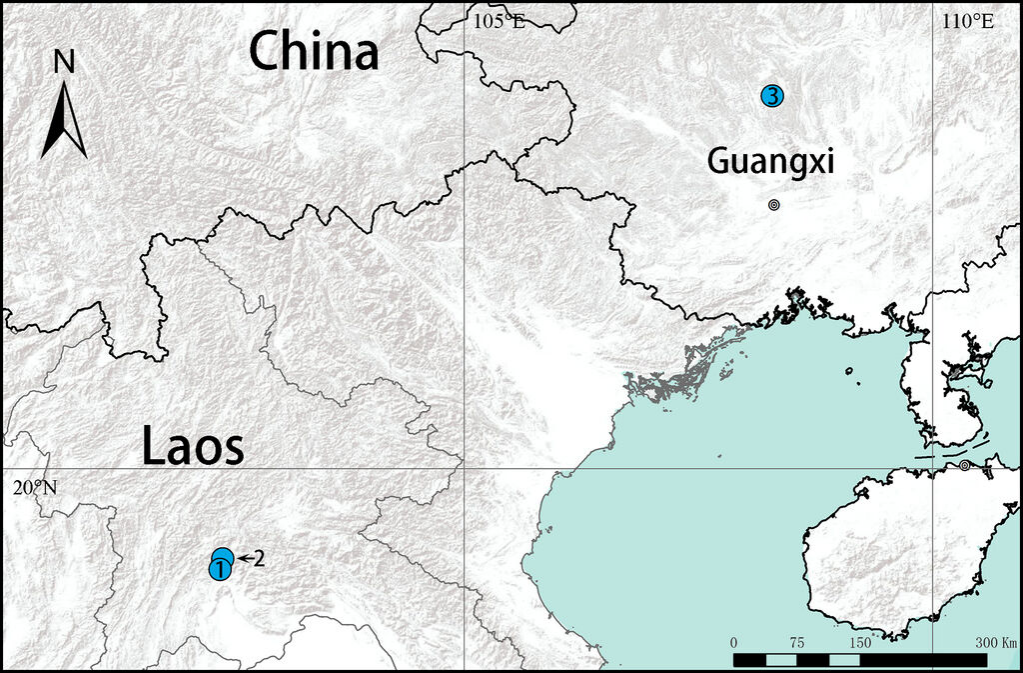
Distribution records of troglobitic *Otacilia* from Asia. **1** *O.saszykaska* Jäger, 2022; **2** *O.tham* Jäger, 2022; **3** *O.khezu* sp. nov.

Known only from the type locality (Fig. 5).

## Supplementary Material

XML Treatment for Otacilia khezu
